# Editorial: Cortical-Subcortical Loops in Sensory Processing

**DOI:** 10.3389/fncir.2022.851612

**Published:** 2022-02-04

**Authors:** Max F. K. Happel, Julio C. Hechavarria, Livia de Hoz

**Affiliations:** ^1^Medical Faculty, MSB Medical School Berlin, Berlin, Germany; ^2^RG CortXplorer, Leibniz-Institute for Neurobiology, Magdeburg, Germany; ^3^Institute for Cell Biology and Neuroscience, Goethe University, Frankfurt am Main, Germany; ^4^Neuroscience Research Center, Charité Medical University, Berlin, Germany

**Keywords:** corticothalamic, cortico-subcortical networks, sensory processing, behavior, neuronal loops, corticofugal, subcortico-cortical

Every organism faces a myriad of sensory stimuli, from which only a small subset is behaviorally relevant to each individual at a given moment. How do brains represent available sensory stimuli in spatiotemporal neuronal activity patterns on one hand, and on the other make the pivotal and immediate selection of information relevant for their individual behavioral responses? It is believed that the hierarchical organization of sensory pathways, from subcortical to cortical structures, allows reciprocal bottom-up and top-down processing of neuronal information *via* the presence of extensive feedback loops between their stations, to select and represent sensory stimuli during processes such as focused perception, attention, and learning (Carandini, 2012). How is this realized within and across different sensory modalities? What specific neural networks, cell-types and neuromodulatory systems play a role in information transmission and selection? In recent years, progress in systems neuroscience has been made, that has allowed us to begin answering some of these questions.

This Research Topic presents a collection of 15 articles that delineate current insights about the commonalities and differences in operational principles of subcortico-cortical loops across sensory modalities, different species, and basic and higher-cognitive functions and brain systems. The collection includes seven original research articles, seven reviews, and one opinion.

In her brief opinion article, Rocklands summarizes the multi-dimensionality of subtypes of corticothalamic neurons, the diversity of microcircuits within and across sensory and neuromodulatory systems, challenging the often-quoted idea of neuronal loops between two areas, or even two directly connected neurons lying significantly apart from each other.

In this line, Mease and Gonzalez aim to connect the predominant single-neuron view of cortico-thalamocortical connections into a broader framework of brain-wide network (dys)functions and their impact on cortical computation, like bursting, and synchronization of ensemble activity. They particularly discuss the interaction of circuits between first-order and higher-order thalamic and cortical regions with respect to function and dysfunction in pain, sensation and cognition.

In this line, Adeyelu et al. provide new evidence that the prevailing view of strictly unilateral thalamo-corticothalamic loops is incorrect. They used retrograde tracing and cre-lox mediated viral anterograde tracing strategies in insular cortex to reveal separate populations of ipsilateral and contralateral projecting corticothalamic layer 6 neurons. These populations also target topographically distinct thalamic subregions.

The topic of higher order thalamic nuclei is also covered by Castejon et al. Here, the authors investigated the function of the posterior medial (POm) nucleus of the thalamus in somatosensory processing. They found that POm is highly sensitive to bilateral multi-whisker stimuli, a finding that challenges the notion of somatosensory thalamus computing only unilateral sensory information. The circuits involved in inter-hemispheric integration involve POm-POm loops formed by thalamocortical and corticothalamic interhemispheric projections. Using the same model but focusing on neuromodulation of feedback processing, Nersisyan et al. found both cholinergic and noradrenergic modulation of L6 projections to POm in rats with somewhat different consequences for repeated stimulation.

In the primary sensory pathways, the corticofugal feedback on subcortical nuclei forms direct cellular response properties of upstream sensory processing neurons. Qi et al. investigated the corticocollicular synaptic transmission from primary auditory cortex (A1) on neurons in the inferior colliculus (ICc). In a detailed study, they systematically mapped corticocollicular input in the ipsilateral ICc to be primarily excitatory and tonotopically mapped between corticocollicular neurons in AI and ICc neurons.

Feedback loops in the auditory system are one of the most studied loops in the brain. In this Research Topic, they are reviewed by Asilador and Llano, Tabas and von Kriegstein, and Antunes and Malmierca from different perspectives. Asilador and Llano provide a thorough review on feedback modulation and predictive coding in the auditory system of humans and animal models. They push forward the idea that corticofugal pathways contain the requisite circuitry to implement predictive coding mechanisms that facilitate the perception of complex sounds, and that different levels of top-down modulation, occurring at subcortical and cortical stages, complement one another. Tabas and von Kriegstein provide a more theoretical perspective on predictive coding. Focusing initially on auditory processing but including later evidence from other modalities across brain regions, they contrast the hypothesis that predictions are computed locally at each processing stage with the more favored hypothesis that predictions are computed globally at higher order stations and then conveyed through feedback projections to lower processing regions. The comprehensive review by Antunes and Malmierca discusses corticothalamic feedback pathways in auditory processing and other sensory modalities. They advance the idea that higher order thalamus could coordinate and contextualize hierarchical inference in cortical hierarchies *via* trans-thalamic pathways.

Loops in the auditory system are also the study topic of Jeschke et al. In this research article the authors developed a small cooling probe to manipulate corticofugal feedback in non-human primates and described changes in cortical and thalamic responses after cortical cooling. Cortical cooling altered spiking dynamics of cortical neurons (i.e., spontaneous activity, cortical spike width), and the temporal and spatial tuning of thalamic neurons.

The role of deep cortical projections in learning is explored by Paraouty and Mowery who found discrimination learning deficits in Mongolian gerbils upon chemogenetic inactivation of L5 auditory cortex projections to the striatum. They further showed that this plasticity is mediated by striatal local inhibition whose levels are tuned during early age, pointing to the likely calibration of subcortico-cortical loops' processing during early sensory experience.

Visual selection is ideal to study sensorimotor integration. A suited readout are eye movements, particularly microsaccades, which are generated by a network of cortical and subcortical neural circuits. Hafed et al. provide a framework on how microsaccades are influenced by peripheral visual cues and impact on visual representation in neurons of superior colliculus and the frontal eye field in primates. The emergent view from stimulation experiments, behavior and theoretical modelling is, that up to date, the visual-motor interactions in perception, attention, and visual acting must be explained by complex cortical and subcortical circuit interactions, which are not fully understood yet. Eye movement research, henceforth, provides an ideal testbed to further differentiate unaddressed reciprocal brain circuits and their impact on complex perceptual, cognitive, and behavioral readouts.

Feedback loops are not only important for information processing in the healthy brain but also mediate how the brain reacts in abnormal conditions, as reviewed by Ewall et al. The authors compiled cortical and subcortical mechanisms that can mediate plasticity upon loss of vision. They summarized potential cellular plasticity mechanisms involved in cross-modal recruitment and compensatory plasticity.

That cortico-subcortical loops exist also between architectonically more complex and diverse circuits than primary sensory circuits, is nicely documented in Lefevre et al., that reviews the reciprocal connectivity of oxytocin neurons in the hypothalamus on various cortical and subcortical structures constituting a brain-wide network to orchestrate social behaviors.

With this Research Topic we contribute to a better understanding of the diversity of these circuits. We should understand neuronal “loops” less as a direct bisynaptic connection between two neurons from different brain areas, but rather as a mutual influence of brain-wide networks, that allow to integrate multi-stranded information from basic sensation, attention, neuromodulation, and action-planning in order to guide adaptive behaviors (Steinmetz et al., 2021).

## Author Contributions

MH, JH, and LH prepared and discussed a list of guests-authors, invited them, revised their manuscripts, and handled their revisions. The Editorial was written by all authors. All authors contributed to the article and approved the submitted version.

## Conflict of Interest

The authors declare that the research was conducted in the absence of any commercial or financial relationships that could be construed as a potential conflict of interest.

## Publisher's Note

All claims expressed in this article are solely those of the authors and do not necessarily represent those of their affiliated organizations, or those of the publisher, the editors and the reviewers. Any product that may be evaluated in this article, or claim that may be made by its manufacturer, is not guaranteed or endorsed by the publisher.

## References

[B1] Carandini M. (2012). From circuits to behavior: a bridge too far? Nat. Neurosci. 15, 507–509. 10.1038/nn.3043 22449960

[B2] Steinmetz N. A. Aydin C. Lebedeva A. Okun M. Pachitariu M. Bauza M.. (2021). Neuropixels 2.0: a miniaturized high-density probe for stable, long-term brain recordings. Science 372:abf4588. 10.1126/science.abf4588 33859006 PMC8244810

